# A Systematic Comparison of the Intrinsic Properties of Wheat and Oat Bran Fractions and Their Effects on Dough and Bread Properties: Elucidation of Chemical Mechanisms, Water Binding, and Steric Hindrance

**DOI:** 10.3390/foods10102311

**Published:** 2021-09-29

**Authors:** Stefano Renzetti, Mira Theunissen, Karlijn Horrevorts

**Affiliations:** 1 Agrotechnology and Food Sciences Group, Wageningen University & Research, Bornse Weilanden 9, 6708 WG Wageningen, The Netherlands; 2 Food Quality and Design Group, Wageningen University, Bornse Weilanden 9, 6708 WG Wageningen, The Netherlands; mira.theunissen@wur.nl (M.T.); karlijn.horrevorts@wur.nl (K.H.)

**Keywords:** bran, bran fractions, water binding, moisture sorption, β-glucan, steric hindrance, breadmaking

## Abstract

This study aimed at elucidating the contribution of chemical interactions, water binding, and steric hindrance on the effect of wheat and oat brans and of their fractions, i.e., soluble and insoluble, on dough and bread properties. For such purpose, an inert filler, i.e., glass beads of comparable particle size and with no water binding capacity and moisture sorption properties, was also studied. The glass beads provided breads most similar to the control, indicating the limited role of steric hindrance. Brans and bran fractions showed distinct compositional and physical properties. The soluble fraction from oat bran, rich in β-glucan, was less hygroscopic than the wheat counterpart and could bind more water, resulting in larger detrimental effects on bread quality. The β-glucan content showed a prevalent role in affecting gluten development, the thermo-setting behaviour of the dough, and crumb texture, i.e., cohesiveness and resilience. Overall, the comparison between the two brans and their fractions indicated that the interplay between water binding, mainly provided by the insoluble fraction, and the plasticizing properties of the soluble bran fraction controlled the effects on bread volume and texture. From a compositional standpoint, β-glucan content was a determining factor that discriminated the effects of wheat and oat brans.

## 1. Introduction

Enrichment of staple food such as bread with cereal brans, a co-product of the milling industry, is raising interest due to consumers and policy makers paying increasing attention to dietary fibre intake and reduction in food waste. Still, the average intake of dietary fibres is generally below the recommended daily dosage [1]. Stimulating the consumption of whole grain products and/or bran-enriched products by improving their perceived attractiveness is the best way to promote higher dietary fibre intake. Cereal brans are rich in dietary fibre, with wheat and oat bran being of particular interest for their nutritional value. Wheat bran includes 37–53% dietary fibre, of which 95% is insoluble fibre, mainly arabinoxylans, cellulose, and lignin [2]. Oat bran contains about 15–20% dietary fibre, of which 61% is soluble fibre, including β-glucan (5–20%) [3]. The health benefits associated with the consumption of wheat bran and oat β-glucan have been recognized by the European Food and Safety Authority in relation to promoting gut health and controlling blood glucose and cholesterol levels [4].

The addition of bran has been associated with detrimental effects on dough and bread properties. Besides the dilution of gluten upon incorporation of bran in bread [5], intrinsic bran properties, as studied in wheat particularly, have been suggested to play a significant role due to interactions with gluten and flour constituents. Interactions between bran and flour are believed to be either physical, chemical, or enzymatic in nature [6]. The mechanisms by which bran negatively impacts dough quality have been ascribed by authors to physical hindrance [7,8], decreased gluten development due to bran competition for water [6,9,10], and by chemical interactions between wheat bran components and gluten proteins which affect gluten network formation [11]. Recently, ref. [10] also showed that the addition of bran affects not only gluten structure but also the thermo-setting behaviour of the dough during heating, shifting starch gelatinization temperatures.

Despite substantial evidence that water redistribution due to fibre addition plays a major role in gluten development [10,12], a combination of mechanisms is most likely to concomitantly act, which extends also to the effect of fibres on starch gelatinization and swelling [13] and on proteins and gluten thermo-setting [14,15,16]. Furthermore, the relationship between the molecular properties of fibres from the soluble and insoluble fractions (SDF and IDF, respectively) and interactions with gluten are not fully elucidated [12]. Wang and co-workers [17,18,19,20] performed a systematic study on the effects of both soluble arabinoxylans (WEAX) and water insoluble solids with a focus on the gluten network and concluded that both fractions had similar effects, driven by the same physical and chemical mechanisms. However, it should be taken into account that while IDF is mainly responsible for water binding and redistribution among food constituents [21], SDF alters the plasticizing properties of the water phase by changes in the volumetric density of hydrogen bonds available for interaction with the biopolymers [13,16]. Hydrogen bonding is also the suggested mechanisms for interactions between fibres and gluten [12,22]. The hydrogen bonding ability of fibres is dependent on the molecular size and the effective number of hydroxyl groups available for interactions [13,16].

While physical and chemical mechanisms largely dictate the effects of bran on dough and bread quality, the specific contribution during different stages of the breadmaking process could substantially vary depending on the bran source and their intrinsic properties [2,23,24]. To the best of our knowledge, there are no studies which addressed a systematic comparison of the effect of wheat and oat bran on dough and bread properties by elucidating the individual contribution of the soluble and insoluble fraction as related to the whole bran. Considering the compositional differences, such comparison could provide further insights into the underlying mechanisms behind bran functionality.

Against this background, the aim of this study was to elucidate the physical and chemical mechanisms by which the soluble and insoluble fractions from wheat and oat brans and their interplay in the whole bran affect dough and bread properties. For such purpose, the physical and chemical properties of the brans and of their soluble and insoluble fractions were characterized for their chemical composition, moisture sorption, and water binding properties and particle size. To better elucidate the mechanisms affecting dough rheology and bread quality, an inert filler, i.e., glass beads with no water sorption and binding properties, was also used. The specific contribution of the bran fractions as compared with the native brans was then studied by replacing the soluble or insoluble fractions previously extracted with the inert filler. In such way, the total amount of “bran” inclusion was kept constant within all variations. The effect of brans on the thermo-mechanical behaviour of the dough and on gluten microstructure was studied by dynamic mechanical thermal analysis and confocal laser scanning microscopy, respectively. These data were then analysed against bread specific volume and crumb texture of the bran-enriched breads.

## 2. Materials and Methods

### 2.1. Materials

Wheat flour Edelweiss (15.5% moisture, 10.6% protein, and 71.2% carbohydrates (of which 1.8% sugars, 2.8% dietary fibres and 95.4% starch)) was from Meneba (Rotterdam, The Netherlands), yeast (Mauripan red) was from AB Mauri (Dordrecht, The Netherlands), the bakery fat trio puur zacht was from CSM Benelux BV (Goes, The Netherlands), and coarse oat bran (5.7% moisture, 18% protein, 33% carbohydrates, 25% dietary fibres, 13.5% β-glucans, 3.50% ash, 0.113% total phenolics as provided by the supplier) of average particle size 300 μm was from ABS Food (Padova, Italy). Coarse wheat bran (13% moisture, 6.5% ash, 15.5% proteins, 38% starch, and 40% dietary fibre as provided by the supplier) was provided by Barilla SpA (Parma, Italy). Glass beads (particle size 200–300 μm) were obtained from HBM (Moordrecht, The Netherlands).

### 2.2. Preparation of Bran Fractions

Wheat and oat bran were first milled to achieve a similar particle size distribution between 200–300 μm using a Cryo miller (6875D, freezer mill, Retsch, Haan, Germany) with a pre-cooling period of 2 min followed by a run time of 2 min. The resulting milled bran was used in the study as native wheat bran (NWB) and native oat bran (NOB). Soluble and insoluble fractions of bran from wheat and oat were obtained according to [25], with some modifications. Briefly, the bran was extracted with demi-water using a bran/water ratio of 3:20 for wheat and 3:40 for oat at room temperature while stirred continuously for 15 min. The samples were separated into a pellet and supernatant by centrifugation at 16,000 rpm for 15 min at 7 °C using an Avanti J-26XP High-Speed Centrifuge from Beckman Coulter (Indianapolis, IN, USA). Samples were freeze-dried, resulting in the insoluble fractions (IWBF for wheat and IOBF for oat) and soluble bran fraction (SWBF for wheat and SOBF for oat). A non-fractionated sample was also prepared by following the same steps but without separation of the supernatant from the pellet. These samples were named reconstituted bran (RWB for wheat and ROB for oat). These sample were included as controls to determine the effect of soaking and freeze-drying on the bran functional properties as compared with the soluble and insoluble fractions and native brans. These samples were also freeze-dried. After freeze-drying, the particles were loosened using a laboratory blender from Waring commercial (Stamford, CT, USA) for 15 s at high speed.

### 2.3. Chemical Characterization of Brans

#### 2.3.1. Moisture Content

The moisture content of the brans and bran fractions was measured in duplicate by drying overnight in aluminium dishes in an oven at 105 °C. The filled dishes were cooled for 1 h in a desiccator before weight determination.

#### 2.3.2. Proximate Analysis

The total dietary fibre (TDF) content was measured in duplicate using the Megazyme Total Dietary Fibre Assay Kit25 (AOAC 991.43, AOAC 985.29, ref. [26]). The protein content was measured in triplicate by combustion (Dumas method, AOAC method 990.03) using a Flash EA 1112 protein analyser (Thermo Fisher Scientific, Waltham, MA, USA). The detected nitrogen content was converted to protein content using 6.25 as conversion factor. The starch content was determined in duplicate according to AOAC (Official Method 996.11) using a total starch assay kit (AA/AMG, Megazyme, Bray, Ireland) according to manufacturer’s instructions.

#### 2.3.3. Total Analysable Carbohydrate Content

Total analysable carbohydrate contents of bran and bran fractions was analysed as follows: (i) free sugars from the water extract, (ii) after hydrolysis with 2 M trifluoracetic acid (TFA) for 1 h at 100 °C (arabinoxylan) of water extract or of samples as such, and (iii) the other carbohydrates were identified after hydrolysis according to [27]. For the water extracts, about 1 g of sample was weighed in a 50 mL Greiner tube, and deionized water was added to reach 25 mL in volume. The solution was then kept under stirring for 2 h at room temperature. After stirring, the solution was centrifuged for 10 min at 16,000× *g*, and the supernatant was recovered. For the WEAX contents, 1 mL of 2 M TFA was added to 1 mL of water extract. For the total arabinoxylans content, 50 mg of sample was weighed in a 50 mL Greiner tube, and 10 mL 2 M TFA was then added. The obtained monosaccharides were determined by high-performance anion exchange chromatography (HPAEC) using an ICS-3000 Ion Chromatography HPLC system equipped with a CarboPac PA-1 column (250 × 2 mm) in combination with a CarboPac PA guard column (25 × 2 mm) and a pulsed electrochemical detector in pulsed amperometric detection mode (Dionex, Sunnyvale, CA, USA) at 20 °C, according to [28]. Analyses were performed in duplicates. Arabinoxylans content was determined as the sum of the arabinose and xylose multiplied by the factor of 0.88 (after correcting for free arabinose and xylose in the water extract before hydrolysis).

#### 2.3.4. β-Glucan Content

The β-glucan content of the wheat flour, of the wheat and oat brans, and of their soluble and insoluble fractions was measured using a mixed-linkages assay kit (AOAC Method 995.16, Megazyme, Bray, Ireland). Briefly, 100–120 mg of flour or bran were treated sequentially with lichenase and β-glucosidase before determination of released glucose by means of the GOPOD reagent. Quantification of glucose was carried out spectrophotometrically (Varian Cary^®^ 50 UV-Vis, Agilent Technologies, Santa Clara, CA, USA).

#### 2.3.5. Free-, Conjugated-, and Bound Phenolics Content

The free/soluble, conjugate soluble, and bound/insoluble phenolics content of the bran samples were determined as follows: for the extraction of free/soluble phenolics, 100 mg of flour was added to 3.75 mL of 80% methanol and mixed for 5 min at 2000 rpm on a vortex, incubated for 30 min at 25 °C at constant agitation of 900 rpm, and the tubes centrifuged at 10,000 rpm for 10 min at 4 °C. The extraction was repeated several times. For the determination of the soluble conjugated phenolic acids, 1.25 mL of the methanolic extract was digested with 0.625 mL of 2 M NaOH for 1 h under nitrogen gas. The hydrolysed samples were acidified with 0.625 mL of 2 M HCl to pH 2. To remove lipid, 1.25 mL of n-hexane was added to the samples, followed by mixing by vortex for 5 min at 2500 rpm, incubation at 25 °C for 10 min with a constant agitation of 500 rpm, and centrifugation for 10 min at 10,000 rpm. After removal of the hexane layer, 1.25 mL of ethyl acetate was added, and the sample was mixed for 5 min at 2500 rpm, incubated at 25 °C for 10 min with a constant agitation of 500 rpm, and centrifuged for 10 min at 10,000 rpm. The ethyl acetate layer (upper phase) was transferred into a 5 mL Eppendorf tube, ethyl acetate evaporated overnight at room temperature, and the samples were resuspended with 1 mL of 80% methanol. Finally, the bound phenolics were determined. Briefly, 1.25 mL of 2 M NaOH in a 5 mL Eppendorf tube was added to the sediment obtained by the extraction of free/soluble phenolics and kept at 90 °C for 30 min under nitrogen gas flush to prevent oxygen degradation. The samples were then vortexed at 2500 rpm for 5 min and acidified to pH 2 with 1.75 mL of 2 M HCl. To remove lipids, 1.25 mL of n-hexane was added to the samples, followed by mixing by vortex for 5 min at 2500 rpm, incubation at 25 °C for 10 min with a constant agitation of 500 rpm, and centrifugation for 10 min at 10,000 rpm. After removal of the hexane layer, 1.5 mL of ethyl acetate was added, and the sample was mixed for 5 min at 2500 rpm, incubated at 25 °C for 10 min with a constant agitation of 5000 rpm, and centrifuged for 10 min at 10,000 rpm. The ethyl acetate layer (upper phase) was transferred into a 5 mL Eppendorf tube, ethyl acetate evaporated overnight at room temperature, and the samples were resuspended with 1 mL of 80% methanol. All the samples were stored at −20 °C before spectrophotometric determination. This was carried out as follows: 375 μL supernatant/standard was added to 300 μL of 25% Folin–Ciocalteu reagent and allowed to react for 5 min. Then, 825 μL of 710 mM Na_2_CO_3_ was added, and the tubes were vortexed at 800 rpm for 10–30 s. The tubes were then incubated at 25 °C for 1 h for colour development in the dark. The solution was then filtered (0.2 μm), and the absorbance was measured at 765 nm against a methanol blank by means of a Varian Cary^®^ 50 UV-Vis (Agilent Technologies, Santa Clara, CA, USA). A calibration curve was prepared in the range of 0–100 μg ferulic acid (FA) per ml, with intervals of 5 μg FA/mL. Results were expressed as FA equivalents.

### 2.4. Bran Physical Characterization

#### 2.4.1. Particle Size Distribution

The particle size distribution of the bran samples (NWB, RWB, IWBF, NOB, ROB, IOBF) was measured in duplicate with the dry powder method of the Malvern Mastersizer 300 from Malvern Panalytical (Malvern, UK). Approximately 2 g of bran was added, with a feed rate of 60%. The air pressure was set to 2 bar, and the machine was set at Opaque Particle type (Fraunhofer Approximation).

#### 2.4.2. Water Binding Capacity

The water binding capacity (WBC) of the bran fractions was determined in triplicate according to a modified version of the protocol of [10]. The bran was soaked in demi-water with a bran/water weight ratio of 1:30. After mixing on a vortex, the samples were left to shake at room temperature for 2 h on a Multi Reax Vortex from Heidolph (Schwabach, Germany). Then, the samples were centrifuged for 60 min at 10,000 rpm using an Avanti J-26XP High Speed Centrifuge from Beckman Coulter (Indianapolis, IN, USA). After discarding the supernatant, the pellet was drained for 15 min at an angle of 45° and then weighed. WBC was expressed as g water taken up per g bran on dry weight basis (DW).

#### 2.4.3. Moisture Sorption Behaviour

The moisture sorption behaviour of the different bran samples was determined in duplicate according to [29], using an automatic multi-sample moisture sorption analyser SPSx-11l from Projekt Messtechnik (Ulm, Germany).

### 2.5. Dough and Bread Preparation and Characterization

#### 2.5.1. Definition of Dough Mixing Conditions

Doughs were prepared by adding 18 g of bran or bran fraction/glass beads mixture or glass beads to 82 g of flour. The level of bran inclusion was chosen as indicative of bran yield in wholemeal flour [30].The bran fraction/glass bead mixtures were prepared following on the ratio of soluble/insoluble fractions determined for the wheat and oat brans (12:88 and 16:84, respectively). Overall, ten different doughs were prepared: a control dough with 100% wheat flour (CB), a control glass bead dough (GB) with 18% glass beads, and the eight doughs containing NWB, RWB, NOB, ROB, SWBF, IWBF, SOBF, and IOBF. To make up for the 18 g of total bran in SWBF, IWBF, SOBF, and IOBF doughs, glass beads of a similar particle size as the milled bran (200–300 μm) were used. The required water absorption for comparable dough consistencies in the bread-baking test was determined in a Farinograph-E (Brabender, Duisburg, Germany) equipped with a 50 g mixing bowl. The ICC standard method 115/1 (ICC-Standards, 2006) was used with few modifications. Briefly, 1 g of sodium chloride (Merck, The Netherlands) was added to 50 g of wheat flour or flour–bran mixtures and pre-mixed for 2 min. Water addition levels were defined by running an appropriate number of replicates until the maximum dough development was centred on the 420 FU (farinograph units), according to a previously established method [10]. The water absorption on 100 g flour as determined by the tests and the corresponding water content in the dough are reported in Table 1. Based on preliminary baking trials, mixing time was kept constant at 9 min for all variations.

#### 2.5.2. Dynamic Mechanical Thermal Analysis (DMTA) of Dough

DMTA tests were conducted in duplicate according to [10] on freshly prepared doughs without yeast. For this, a DHR-2 Rheometer from TA Instruments (New Castle, DE, USA) was used with Peltier plates of parallel geometry and a diameter of 25 mm. Approximately 1 g of dough was placed between plates (loading gap: 20 mm) and compressed until 1.025 mm. Dough excess was removed, silicon oil was applied to prevent sample drying, and dough was compressed until 1 mm. Before the measurement, the dough was rested for 5 min at 25 °C. Samples were oscillated at a frequency of 1 Hz and heated from 25 to 120 °C with a ramp of 5 °C/min. Before analysis, an oscillation amplitude test was performed from 10^−4^ to 10 to select the linear viscoelastic range. Thus, the strain amplitude was kept at 0.5·10^−3^ for all samples. Key parameters related to physical transitions in the dough were derived from the analysis of the G′ (Pa) and tan(δ) curves in the DMTA by using the analysis functions in TA Trios v3.3 (TA Instruments, New Castle DE, USA): the G’ and tanδ value at 25 °C; the onset temperature, T_onset_, from the evolution of G′ during heating (calculated as the intersection of the tangents of the baseline before the sudden increase in G′ and the tangent of the steep G′ profile after T_onset_); G′ at maximum (G′_max_); and the temperature corresponding to G′_max_, i.e., T_peak_.

T_onset_ and T_peak_ from the DMTA analysis were plotted in the state diagram for bread dough baking, as recently constructed by applying an adapted Flory–Huggins model [13,30]. The volume fraction of water in each dough was calculated based on the flour (or flour/bran mixtures) and water contents (Table 1) and mass densities, as previously described [13,16].

#### 2.5.3. Confocal Laser Scanning Microscopy of Dough

To visualize the gluten network in the dough matrix, confocal laser scanning microscopy (CLSM) was performed based on the method of [31], with slight modifications. Doughs without yeast were prepared, in which water was partially substituted with a filtered Rhodamine B solution in demi-water (0.1 g/L) to reach a concentration of 1 mg Rhodamine B per 100 g flour. Per dough type (in duplicates), ten images were taken with a LSM 510-META CLSM from Zeiss (Jena, Germany) using 20× magnification and a resolution of 1024 × 1024 pixels. To get quantitative results, the images were analysed with AngioTool64 version 0.6a from the National Cancer Institute (Maryland, MD, USA) (low threshold: 15; high threshold: 255; vessel thickness: 9, 20; small particles: 10; fill holes: 0 and scaling factor: 0.0006640625). Parameters of interest included total protein area, total number of junctions, average protein length, protein branching rate (total number of junctions/total protein area), and average protein width (protein area/total protein length).

#### 2.5.4. Bread Baking and Baking Quality Evaluation

Breads were prepared in triplicate for each variation according to the method used by [10]. In order to eliminate the effect of compositional variations on yeast metabolism, the final fermentation time was defined as the time needed to produce 120 mL of CO_2_, as measured in a Risograph from National Manufacturing (Lincoln, RI, USA). Baking took place in a custom-made swing oven at 230 °C for 20 min. Steam was injected twice at the start of each bake. The breads were cooled for 60 min, packed in plastic bags, and evaluated the day after. Loaf volume was determined on 4 loaves for each variation, with a rapeseed displacement according to the AACC method 10–05.01. Specific volume was calculated as loaf volume divided by loaf weight (mL/g).

#### 2.5.5. Crumb Texture and Moisture Content

Crumb texture was measured by means of Texture Profile Analysis using a TA-XT2i Texture Analyser from Stable Micro Systems (Godalming, Surrey, UK) with a 5 kg load cell and a 75 mm compression plate and performed as described by [10]. Twelve independent measurements were performed per bread type. The moisture content of the bread crumbs (5 g sample) was measured in triplicate in a similar way as for the brans.

### 2.6. Statistical Analysis

Statistical evaluation (analysis of variance, ANOVA, with Tukey’s test as the post hoc test, at a significance level of *p* < 0.05) was performed with SPSS (IBM, version 25, Chicago, IL, US); principal component analysis (PCA), correlation analysis, and regressions were performed with RStudio (RStudio version 1.1.463, Inc., Boston, MA, USA).

## 3. Results

### 3.1. Chemical Composition and Physical Properties of Brans and Their Fractions

The chemical composition and physical properties of wheat and oat brans and their fractions are reported in Table 2. The chemical composition of the NOB was similar to the supplier’s specifications; while for NWB, lower TDF and starch contents and higher protein content were measured compared with specifications. Oat bran (NOB, ROB) was richer in starch and proteins compared with wheat bran (NWB, RWB) (*p* < 0.05). NWB contained more TDF than NOB (*p* < 0.05). The chemical composition of brans after hydration and freeze-drying (ROB and RWB) was similar to the native brans (NOB and NWB, respectively), except for a significant increase in WEAX and reduction in starch content for RWB as compared with NWB (*p* < 0.05).

For wheat bran, IWBF showed the highest TDF content, while for oat bran the highest TDF was found in SOBF (*p* < 0.05). SWBF showed the lowest TDF content (*p* < 0.05). TDF in SOBF was mainly β-glucans. Consequently, β-glucans were significantly lower in IOB compared with NOB (*p* < 0.05). Wheat bran fractions were richer in AX compared with oat bran fractions (*p* < 0.05). SWBF showed the highest amounts of WEAX (*p* < 0.05). Protein content was found the highest in SWBF (*p* < 0.05). Starch content was the highest in IWBF and IOBF as a result of the fractionation, while SWBF and SOBF had the lowest starch content (*p* < 0.05).

The total phenolics content was higher in NWB and RWB than in NOB and ROB (*p* < 0.05). For wheat bran, the highest amount of phenolics was found as bound phenolics. On the contrary, the soluble fractions SWBF and SOBF showed the highest amounts of free phenolics.

**Figure 1:**
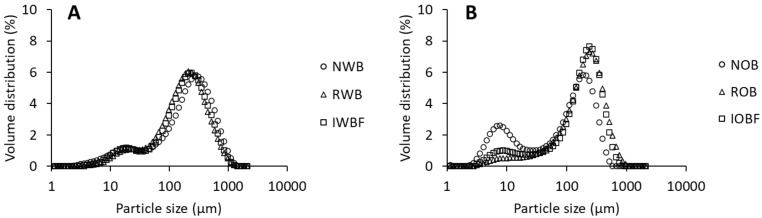
Particle size distribution of native brans, reconstituted brans, and insoluble fractions from wheat (**A**) and oat (**B**). Sample abbreviations are reported in Table 1.

With regard to physical properties, the particle size distribution of brans showed a similar profile, with a peak between 200 and 300 μm (Figure 1), although a small peak of particles around 10 μm was observed for NOB. As intended, the particle size of the brans was within the range of the glass beads. NWB showed the highest volume weighted mean diameter, i.e., D_[4,3]_, while NOB showed the lowest (*p* < 0.05) (Table 2). No significant differences were detected for RWB, IWBF, and IOBF. The water binding and moisture sorption behaviour of the brans and their fractions were also characterized, as they are an important aspect of their technological functionality. NOB showed a higher WBC compared with NWB (*p* < 0.05). NOB and SOBF had the overall highest WBC. For SOBF, the observed WBC resulted from a gel that formed after hydration and centrifugation. On the contrary, the SWBF did not have any water binding ability, as no pellet could be observed after centrifugation. Additionally, as expected, the glass beads were not able to bind any water.

**Figure 2:**
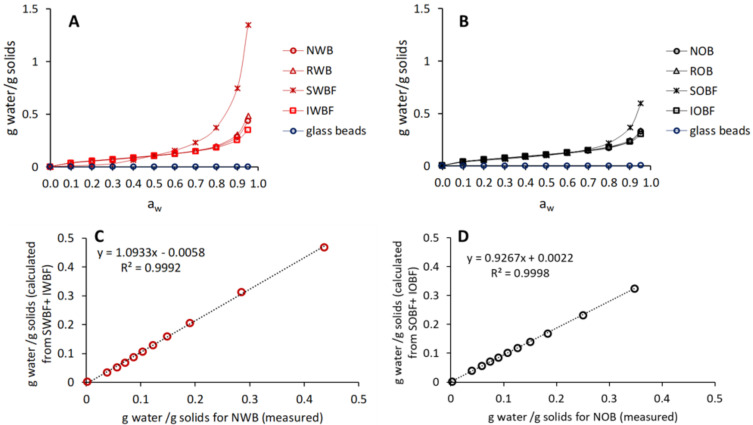
Moisture sorption behaviour of wheat bran (**A**) and oat bran (**B**) and their fractions as compared with glass beads. Moisture sorption computed from the isotherms of the soluble and insoluble bran fractions as compared with the native wheat (**C**) and oat (**D**) brans. Sample abbreviations are reported in Table 1.

The moisture sorption behaviour of the different brans as well as glass beads is shown in Figure 2A,B. Overall, NWB was able to adsorb more water than NOB, as also shown by the water which was adsorbed at an a_w_ of 0.95 (Table 2). The different behaviour between NWB and NOB, as well as their reconstituted fractions, could be largely related to the differences among the soluble fractions of the two bran sources. Clearly, SWBF was considerably more hygroscopic than SOBF (*p* < 0.05), while IWBF and IOBF were the two fractions with the lowest adsorption behaviour (Table 2). The determining contribution of the soluble fractions to the sorption behaviour of the wheat and oat brans was also confirmed by computing the sorption behaviour of the native brans from the soluble and insoluble fractions based on the mass fraction obtained during the fractionation experiments (for NWB: SWBF = 0.12 and IWBF = 0.88, for NOB: SOBF = 0.16 and IOBF = 0.84). As shown in Figure 2C,D, the computed values were in good agreement with the measured sorption isotherm.

### 3.2. Dough Thermo-Mechanical Properties during Temperature Sweep

**Figure 3:**
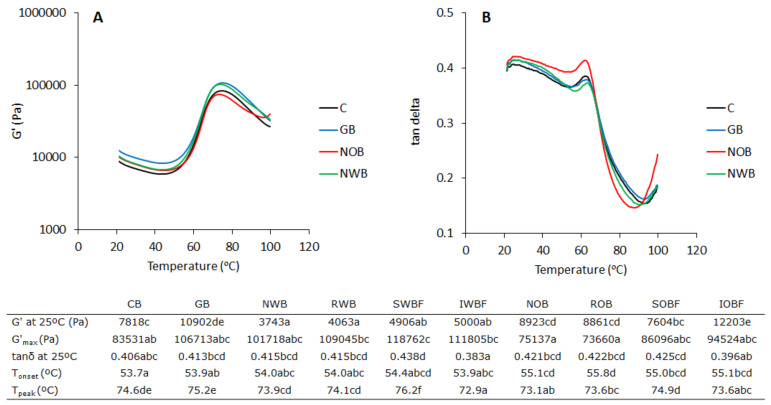
Examples of DMTA profiles for wheat dough enriched with glass beads and native wheat (NWB) and oat (NOB) brans showing the evolution of G’ and tanδ as a function of temperature. The table shows the parameters extracted from the curves for all dough variations. Different letters in the same row indicate statistically significant differences (*p* < 0.05). Sample abbreviations are reported in Table 1.

The thermo-mechanical behaviour of the different wheat doughs was investigated by DMTA. The technique provides insights into the visco-elastic properties of the dough (G’ and tan δ) as affected by key phase transitions such as gluten thermosetting and starch gelatinization. These transitions largely control the structure formation during baking of dough and other cereal-based matrices [14,16,29,32]. In general, three main phases can be observed with regard to the changes in G’ as a function of temperature (Figure 3). A decrease in G’ going from 25 °C to 50 °C, approximately, is associated with softening of the dough. A sharp increase in G’ in the temperature range between 50 and 60 °C is associated with the beginning of structure setting, i.e., T_onset_. A further increase in G′ is observed beyond T_onset_, reaching a maximum, i.e., G′_max_ around 70–75 °C, after which a typical decrease in the gel strength is observed with increasing temperature [33,34].

The addition of all wheat bran fractions resulted in a significant reduction in G’ at 25 °C compared with the control (*p* < 0.05) (Figure 3). This was opposite to the effect of glass beads, which resulted in a significant increase in G’ at 25 °C (*p* < 0.05). Among the oat bran fractions, only IOBF showed a significant increase in G’, which was similar to the dough with glass beads. Overall, it should be noted that the addition of glass beads and of wheat and oat brans (NWB, RWB, NOB, ROB) seemed to enhance more the viscous behaviour over the elastic one compared with the control, as described by tanδ at 25 ºC, despite finding that the effect was not significant (Figure 3). Distinct and significant effects on tanδ could be observed when comparing the soluble and the insoluble fractions. While SWBF and SOBF increased tanδ, IWFB and IOBF decreased it. These opposite effects for soluble and insoluble fractions were significant for the tanδ at 25 °C (*p* < 0.05). For the dough enriched with SWBF, the tanδ at 25 °C showed the overall highest values.

**Figure 4:**
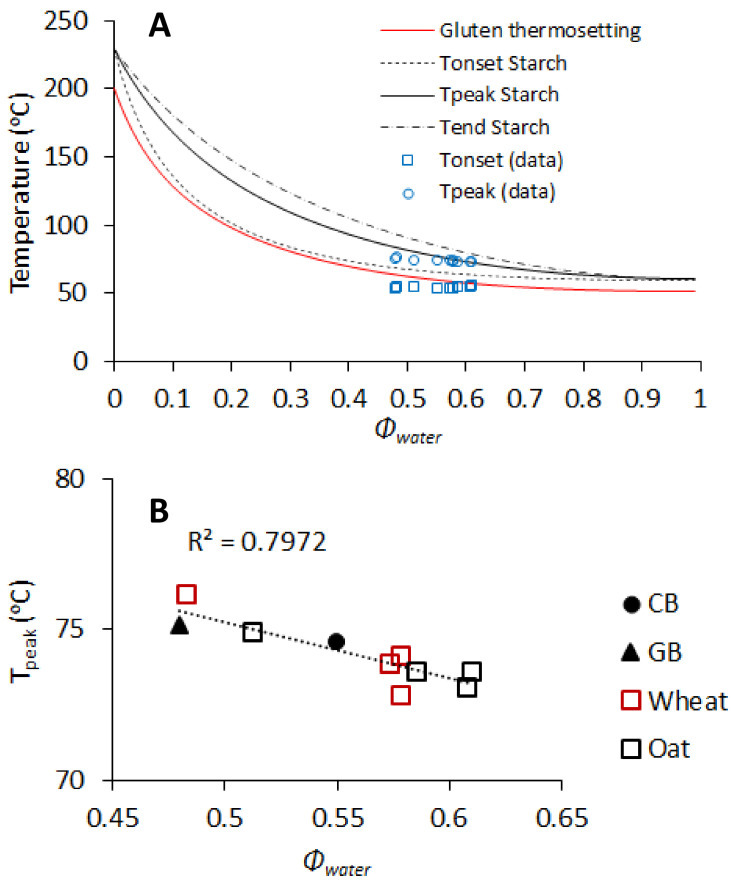
T_onset_ and T_peak_ as determined for the different dough samples from DMTA plotted in the supplemented state diagram for dough baking with phase transitions for gluten and starch (**A**). The state diagram is adapted from [13,14]. Correlation between T_peak_ and the volume fraction of water Φ_water_ in each dough (**B**). CB = control dough and GB = dough with glass beads.

Glass beads and the wheat bran fractions did not have a significant effect on T_onset_, while all oat bran fractions significantly increased it (*p* < 0.05) (Figure 3). Overall, the increase was within 2 °C. Larger effects were observed for T_peak_. SWBF showed a significant increase in T_peak_, while IWFB, NOB, ROB, and IOBF decreased it (*p* < 0.05). The addition of glass beads alone had no effects compared with the control dough. The T_onset_ has been often associated with the onset of starch gelatinization [33,34], as a high correlation can be found between the T_onset_ in DMTA and the onset of starch gelatinization from DSC analysis [10]. However, a contribution from gluten thermosetting cannot be ruled out, as these two transitions occur at around similar temperatures, as recently reported [14,35,36]. The T_peak_ corresponding to the G′_max_ has been associated with the peak gelatinization temperature T_peak_ [10]. To further elucidate what was controlling the structuring transitions, T_onset_ and T_peak_ were plotted in the state diagram for bread dough baking (Figure 4A). The state diagram was constructed using the gluten thermosetting and starch melting curves as a function of Φ_water_, which we previously reported based on applying an adapted Flory–Huggins model [13,14]. As it can be observed, both T_onset_ and T_peak_ were well in agreement with the predictions from the supplemented state diagram. These transitions were largely controlled by the amount of water present in the dough, as shown by the correlation between the T_peak_ and volume fraction of water in the dough Φ_water_ (*p* < 0.05) (Figure 4B).

With regard to G’_max_, only SWBF showed a significant effect as compared with the control dough, enhancing the elastic modulus (*p* < 0.05). This effect was quite remarkable considering that a small amount of the fraction was added.

### 3.3. Protein Network

**Figure 5:**
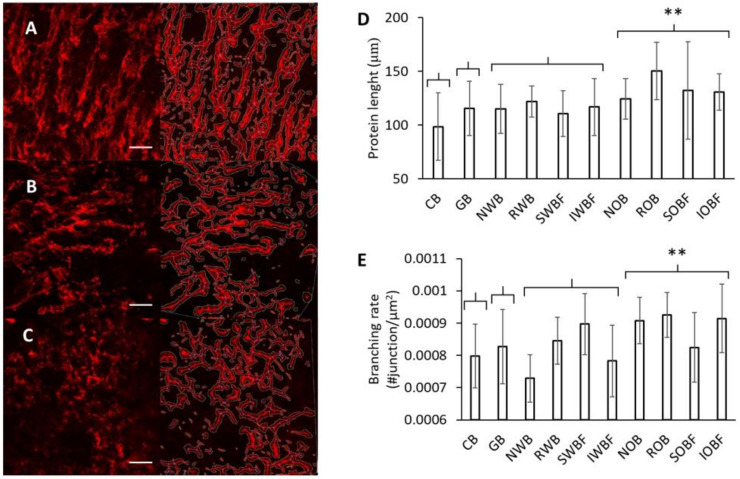
Examples of gluten microstructure visualized for the control dough (**A**) NWB-enriched dough (**B**) and NOB-enriched dough (**C**). The resulting analysis of the image using the Angiotool software is shown on the right for each sample. Protein length (**D**) and branching rate (**E**) as determined for all dough samples using the Angiotool. The symbol (**) indicates a significant (*p* < 0.05) effect of the bran source. The white scale bar in (**A**–**C**) indicates 100 μm. Sample abbreviations are reported in Table 1.

CLSM was performed on all doughs in order to visualize changes in the gluten network, as affected by addition of glass beads and bran fractions (Figure 5A–C). In general, the addition of bran and bran fractions from wheat and oat seemed to affect the typical protein strands observed in the control dough. Image analysis was performed to quantify these differences using the Angiotool software, as previously reported [31]. Despite the large variations observed in the analysis of the network, significant differences were observed on the impact of the bran sources (wheat, oat, or glass beads) on the average protein length and branching rate (Figure 5D,E). In fact, the addition of oat bran and its fractions resulted in a significant increase in protein length and branching rate as compared with the control (*p* < 0.05). Similarly, substantial changes in the protein network were recently observed with the addition of fibre-rich ingredients such as oat β-glucan [37], with protein length and branching rate significantly increased in the presence of bran [38]. The addition of glass beads and wheat bran fractions also caused an increase in protein length, although the effect was not significant.

### 3.4. Bread Quality

**Figure 6:**
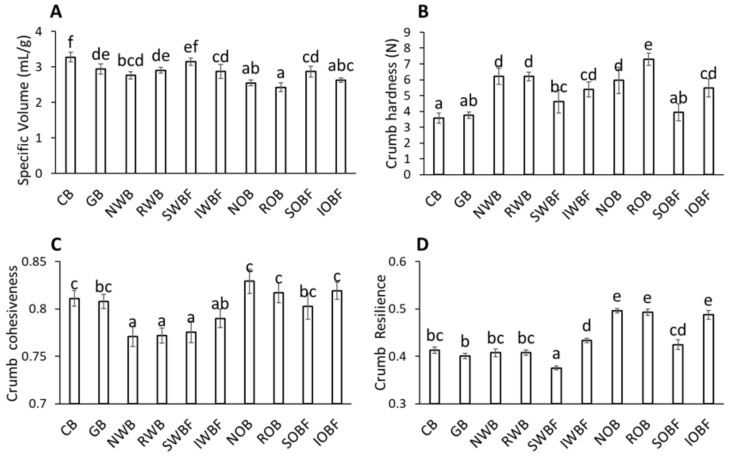
Bread quality parameters for breads obtained with enrichment in bran and bran fractions and glass beads: specific volume (**A**), crumb hardness (**B**), crumb cohesiveness (**C**), and crumb resilience (**D**). Sample abbreviations are reported in Table 1. Different letters in the bar charts indicate statistically significant differences (*p* < 0.05).

The addition of glass beads and bran fractions had significant effects on bread quality (Figure 6). Specific volume was the highest for CB and SWBF, while the addition of glass beads and the other bran fractions all resulted in a significant reduction in specific volume compared with the control (*p* < 0.05). The addition of oat bran and its fractions showed the largest detrimental effect on specific volume (*p* < 0.05), while the effects of glass beads and wheat bran fractions were comparable.

With regard to texture, CB and GB breads showed the softest crumb (*p* < 0.05), while wheat and oat bran fractions provided a significant increase in crumb hardness (*p* < 0.05). Among the bran-enriched breads, only SOBF had a similar hardness as CB and GB breads. Chewiness and gumminess showed similar trends as for hardness (data not shown). In terms of cohesiveness, the breads enriched with wheat bran fractions showed a significant reduction compared with the control (*p* < 0.05). On the contrary, the addition of glass beads and oat bran fractions did not show significant effects on cohesiveness as compared with the control. The SWBF bread showed a significantly lower resilience than CB (*p* < 0.05), while that of IWBF, NOB, ROB, and IOBF breads was significantly higher (*p* < 0.05).

### 3.5. Relation between Chemical and Physical Properties of Brans, Dough Properties, and Bread Quality

**Figure 7:**
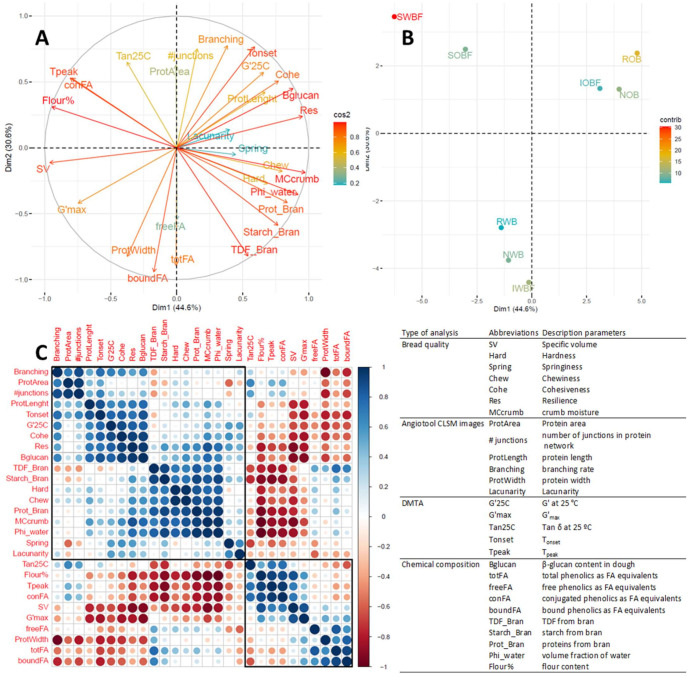
PCA analysis with loading plot of chemical, rheological, microstructural, and bread quality parameters (**A**) and samples score plot (**B**). Map of correlations analysis between the different parameters (**C**). The table provides information on the type of analysis, abbreviation, and description for each parameter. Additional details for protein network parameters are provided in [31]. Sample abbreviations are reported in Table 1.

A PCA analysis was performed on wheat and oat bran enriched breads to better distinguish the effect of the bran sources and their fractions. For such purpose, the contribution of the bran fraction to the dough composition was computed based on dough recipe and chemical analysis of brans. The rheological properties of the dough, the gluten microstructure data, and the properties of the resulting breads were also included in the analysis. The sample variations (score plot) and the chemical, rheological, microstructural, and bread quality parameters (loading plot) are shown in Figure 7A,B, respectively, on the plane PC_1_–PC_2_. The first two principal components accounted for 75.2% of the variance, with the other PCs contributing less than 14% each; therefore, it is possible to consider this representation as very indicative of the differences between the bran-enriched samples. Three distinct groups could be observed by their separation in the score plot of the PCA: the soluble fractions SWBF and SOBF in the IV quadrant, the oat bran (NOB and ROB) and its insoluble fraction (IOBF) in the I quadrant, the wheat bran (NWB and RWB), and its insoluble fraction (IWBF) in between quadrant II and III. NOB, ROB, and IOBF were characterized by a high β-glucan content in the dough, high protein length and branching rate of the gluten network, high T_onset_ of structure setting, and high cohesiveness and resilience of the crumb. Breads from NOB, ROB, and IOBF also showed the lowest specific volumes of the breads. NWB, RWB, and IWBF were characterized by high phenolics contents in the dough. SWBF and SOBF were characterized by the highest flour content in the dough (and correspondingly the lowest volume fraction of water) and high T_peak_. The bread crumbs from these fractions also had the lowest hardness.

**Figure 8:**
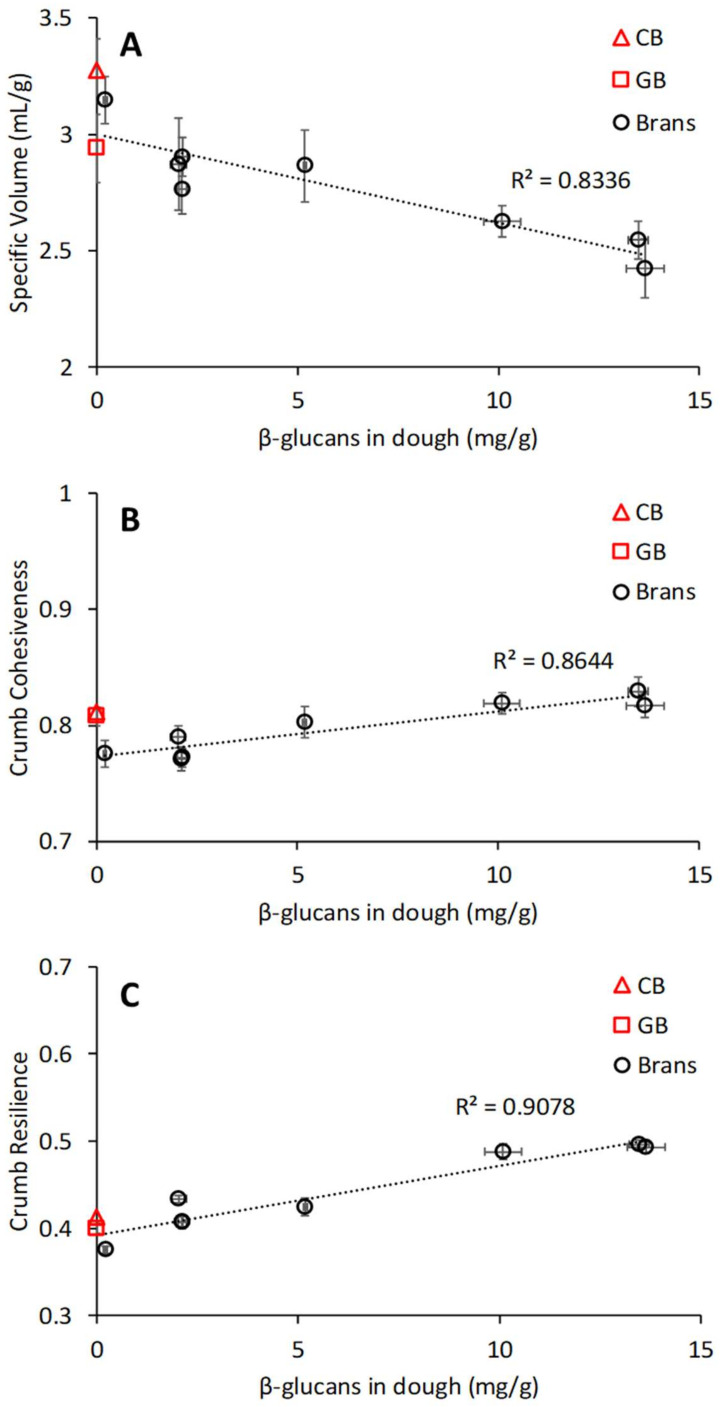
Association between the β-glucan contents in the dough and some bread quality parameters: specific volume (**A**), crumb cohesiveness (**B**), and crumb resilience (**C**). CB = control dough and GB = dough with glass beads.

**Figure 9:**
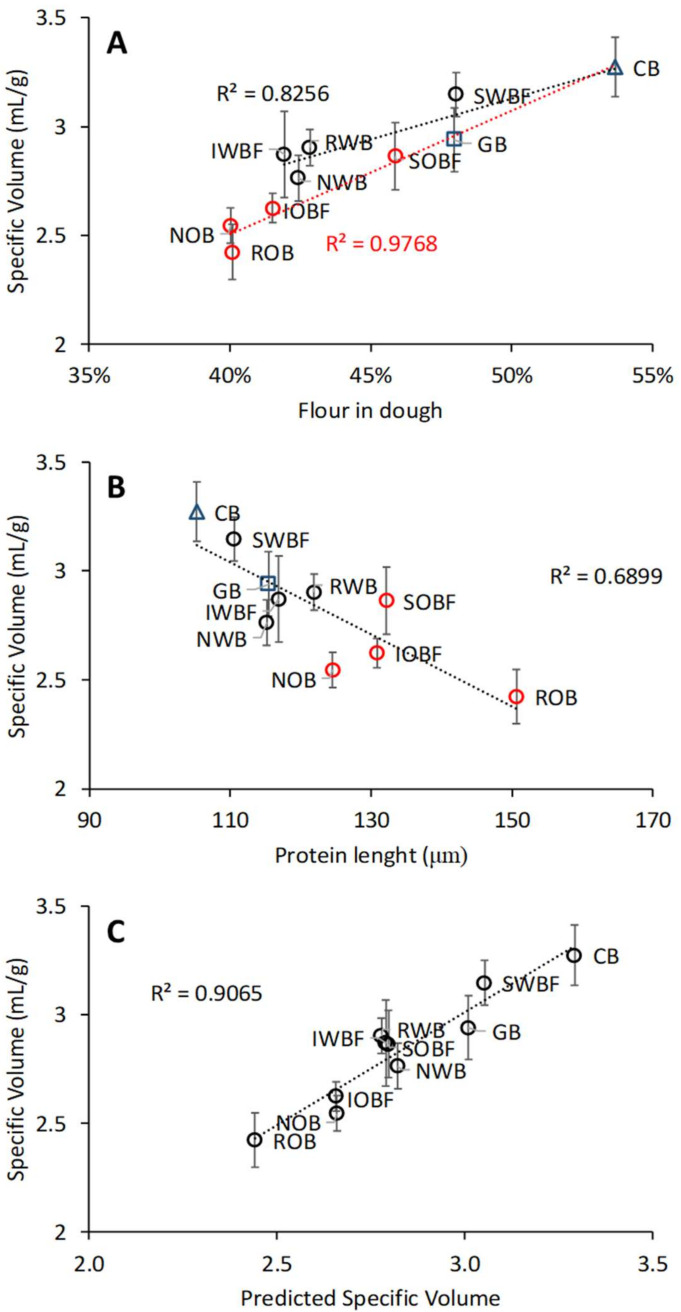
Association of specific volume with flour content in the dough (**A**) and with protein length as determined by analysis of CLSM images (**B**). Correlation between measured specific volume and the one predicted from flour content and protein length (**C**). In figures (**A**,**B**), black circles indicate wheat brans, and red circles indicate oat brans. Sample abbreviations are reported in Table 1.

A correlation analysis among the variables was performed, as shown in Figure 7C. Among the most relevant associations were the positive correlations of the β-glucan content in the dough with crumb cohesiveness and resilience (R^2^ = 0.864 and 0.908, respectively, *p* < 0.001) and between bread specific volume and flour content in the dough (R^2^ = 0.768, *p* < 0.001). Inverse associations were instead found for specific volume with protein length and β-glucan content in the dough (R^2^ = 0.582 with *p* < 0.05 and 0.836 with *p* < 0.001, respectively). Furthermore, β-glucans content negatively correlated with the maximum gel strength G′_max_ of the dough during heating in the DMTA (R^2^ = 0.83, *p* < 0.05). The predominant effect of β-glucan content on bread quality (i.e., specific volume, crumb cohesiveness, and resilience) is clearly shown in Figure 8A–C as compared with CB and GB breads. A further analysis of the data revealed that the specific volume showed distinct dependence on flour content, depending on the source of bran fractions (wheat or oat), as shown in Figure 9A. The oat bran fractions enhanced protein length as obtained from the analysis of CLSM images, resulting in lower specific volume than their wheat counterparts (Figure 9B). A linear regression analysis indicated that the specific volume could be well described for all variations by the combined effect of flour content and protein length (Figure 9C), with both parameters having a significant contribution (*p* < 0.001).

## 4. Discussion

Chemical and physical mechanisms have been suggested as the main factors controlling the functionality of bran in bread applications, although their impact on bread quality is still not fully elucidated. For wheat bran, these mechanisms have been summarized in physical hindrance, competition for water, and chemical interaction of gluten with ferulic acid, arabinoxylans, and glutathione [6]—all of them resulting in detrimental effects. However, there are limited studies which have systematically compared these mechanisms between different bran sources and bran fractions, i.e., soluble and insoluble. In particular, the interplay between the effect of the soluble and insoluble fractions as compared with the whole bran have not been elucidated. In this study, we investigated the effect of two bran sources with different compositions, i.e., wheat and oat, and of their fractions, i.e., soluble and insoluble, on dough and bread properties with the aim of further elucidating the interplay of chemical and physical mechanisms from the different bran fractions. For such purpose, the effect of brans was also compared with that of an inert filler, i.e., glass beads, of comparable particle size, which did not have any water binding capacity and water adsorption properties (Figure 2) as compared with the brans and their fractions. The glass beads were also used to compensate for the missing fraction of the brans when only the soluble or insoluble extracts were added.

The compositional and physical characteristics of wheat and oat brans, and their corresponding fractions, varied significantly (Table 2), in line with the ingredients specifications. The proximate composition of NWB was in agreement with ranges recently reported for regular bran [6]. Protein content in NOB was in agreement with ranges reported by [39], while its TDF content was higher than the 19.7–20.6 (% of dry matter) reported by [40], with a concomitant lower starch content. This can probably be due to differences in fractionation procedures. The total AX and WEAX contents in NWB was according to ranges recently reviewed by [41], while β-glucan content was within ranges reported by [6]. The total AX content in NOB was similar to what was earlier reported [40,42]. Wetting and successive freeze-drying of the native wheat and oat brans, NWB and NOB, respectively, did not alter the chemical composition except for a significant increase in WEAX and reduction in starch content for RWB as compared with NWB. The action of endogenous endo-xylanases and α-amylases during wetting could explain such differences [43]. Indeed, total sugars in the water extract (i.e., free sugars) and after TFA hydrolysis were higher for RWB than NWB, with a particular increase in glucose contents (data not shown), which likely originated from starch degradation. It should also be noted that not all material could be recovered from the total sum of the proximate analysis, with largest deviations for the soluble fractions and for RWB. As the TDF determination method employed did not account for the low molecular weight dietary fibres [44] and because endogenous enzymatic activities likely occurred during the wetting and extraction procedures, it can be suggested that these factors contributed to the observed differences, with SWBF and RWB being the most affected.

With regard to the physical properties, the WBC of NOB was the highest among all bran fractions and in agreement with values previously reported [45]. Wetting and freeze-drying significantly reduced the WBC of ROB as compared with NOB. Concomitantly, SOBF showed a WBC similar to NOB. The ability of soluble dietary fibres from oats to bind a high amount of water has been previously reported [46]. Since the water binding ability of β-glucans is dependent on molecular weight, it can be suggested that endogenous β-glucanase activity during preparation of ROB may have affected its WBC [47].

The moisture sorption behaviour differed significantly between wheat and oat brans. Despite the fact that NWB had a lower soluble fraction content than NOB (SWBF being 12% and SOBF being 16% of the corresponding native brans), NWB and RWB showed a more hygroscopic behaviour than NOB and ROB, in agreement with previous findings [48]. It has been previously reported that for many carbohydrates, including soluble fibres and soluble polymeric glucose, and for proteins, the sorption behaviour is affected by the average molecular weight [49,50,51]. The concept also applies to mixtures of carbohydrates and proteins [52], as it did in the case of the brans and bran fractions in this study. Small molecules are able to adsorb more water than larger ones. SWBF showed the highest adsorption of water at 95% RH (*p* < 0.05), which was 2.25 times that of SOB and 3.85–4.5 times that of the insoluble fractions IWBF and IOBF, respectively (Table 2). Hence, it can be suggested that the SWBF was composed of polymers considerably lower in Mw than SOBF, thus explaining the differences in sorption behaviour. These differences among the soluble fractions largely impacted the sorption behaviour of the native brans (NWB and NOB) and the reconstituted ones (RWB and ROB) (Figure 2). In fact, the differences between NWB and NOB were larger than between IWBF and IOBF (Table 2).

In the attempt to distinguish the contribution of chemical and physical properties of brans and bran fractions to dough and bread quality, glass beads were also used as inert fillers. Since the results suggest that they are physically and chemically inert, their use elucidates the limited role of steric hindrance and gluten dilution on dough and bread quality. In fact, their addition to dough showed the least detrimental effects regarding SV and crumb texture. Specific volume of GB bread was reduced by only 10% when compared with the control, while no differences were observed in terms of crumb hardness, cohesiveness, and resilience. Using a synthetic bran with no water binding ability, ref. [7] also showed a limited effect on the SV of bread, but its moisture sorption behaviour and the impact on crumb texture was not elucidated. Considering that the glass beads and the brans had a similar particle size distribution, it can be concluded that differences in water binding and sorption properties and in chemical compositions among the brans and bran fractions were mainly responsible for the observed differences in dough and bread characteristics, rather than steric hindrance. The CLSM investigation of the gluten network, showing the very similar characteristics of the control dough and the GB dough, further supports this hypothesis. From such a standpoint, the use of the glass beads to compensate for the missing fractions was a valid approach to systematically distinguish the contribution of the soluble and insoluble fractions alone.

Recently, the adverse effects of bran addition to bread have been predominantly related to its water binding capacity and hydration behaviour [7,43,53]. However, the results of this study indicate that for a better understanding of bran functionality, it is important to consider both the water binding properties (intended as the ability to retain water) and the plasticizing properties of the soluble bran fractions (intended as the ability to change the structure and properties of the water phase). In fact, RWB and ROB showed similar WBC and average particle size (Table 2), but the amount of water required in the dough to ensure proper development was considerably higher for the ROB-enriched one (Table 1). The SV of ROB was also significantly lower than the RWB-enriched bread. On the contrary, the insoluble fractions IWBF and IOBF provided breads which were close in SV and in textural properties (Figure 6), while the differences between the whole wheat brans (NWB and RWB) and whole oat brans (NOB and ROB) were significantly larger. These results suggest an important contribution of the soluble bran fractions to the functionality of the native and reconstituted brans with regard to the interplay between water binding and plasticizing ability. This was also pointed out by the significant differences between the soluble and the insoluble fractions on dough rheology. In fact, SWBF and SOBF enhanced viscous behaviour (higher tanδ at 25 °C), while IWBF and IOBF enhanced elastic behaviour (lower tanδ at 25 °C) (Figure 3), in agreement with what was recently reported [36]. Aside from the water binding exerted by the insoluble bran fractions, the presence of solutes in the water phase affects the structure of water, as hydrogen bond interactions between solutes and water result in a reduction in bulk water and the formation of a hydration shell [54], with the dynamics of water in the hydration shell being slower than in the bulk. When looking at the soluble bran fractions, the SWBF-enriched dough required much less water than the SOBF-enriched dough, even lower than the dough with glass beads only (Table 1). The SWBF-enriched dough also had the highest tanδ at 25 °C, indicative of enhanced viscous moduli compared with the elastic one. In view of the explained moisture sorption behaviour of SWBF as compared with SOB, it can be suggested that the smaller molecules in SWBF acted as better plasticizers than those in SOBF. We have recently reported that a wide range of solutes (i.e., sugars, amino acids and small peptides, soluble fibres) can indeed act as plasticizers for proteins, including gluten, and for starches [13,16,30,55]. Polysaccharides with low Mw (and hence low molar volumes) and a high number of H-bonding sites act as better plasticizer than larger ones. The lower plasticizing ability of SOBF compared with SOBF, combined with the high water binding capacity, likely contributed to the significant differences observed between wheat bran (NWB and RWB) and oat bran (NOB and ROB) enriched breads (Figure 6).

The interplay of water binding and plasticizing properties governed the water adsorption determined by farinograph. This affected not only dough development but also the baking behaviour of the dough. In fact, the volume fraction of water, Φ_water_, could largely explain the structure setting behaviour of the dough during heating in the DMTA, as demonstrated by the good representation of T_onset_ and T_peak_ in the supplemented state diagram (Figure 3). Gluten thermosetting and starch gelatinization occurred at lower temperatures with increasing Φ_water_, as was the case for the oat brans compared with their wheat counterparts. An earlier structure setting can contribute to lower loaf volume, although it is clearly not the only factor, with gluten development playing a determining role.

The differences in water absorption by the flour and flour/bran mixtures resulted in differences in flour content in the dough. Variations in flour content could only partially explain the effect on bread quality, as pointed out by the distinct trends for wheat and oat fractions with regard to SV (Figure 8A). Compared with wheat, oat and oat bran fractions had a larger effect on gluten microstructure, as pointed out by the differences in protein length and branching rate (Figure 5). In particular, changes in protein length significantly correlated with SV (Figure 9B). As recently reported [10], bran addition hinders the development of a gluten secondary structure that is optimal for baking. The rheological properties of the dough most relevant for breadmaking, particularly the strain hardening behaviour, are negatively impacted by bran enrichment with significant changes in protein length and branching rate in the gluten microstructure [38]. Overall, the combined changes in flour content and gluten network structure best captured the observed variations in SV (Figure 9C). The contribution from flour content may well relate to both the gluten and the starch components, as both the gluten thermosetting and starch gelatinization and swelling contribute to dough rheology during baking [56].

A PCA of bran-enriched dough/bread variations and a correlation analysis of dough composition and dough and bread properties were performed to further elucidate the contribution of chemical mechanisms. As earlier discussed, flour and water content, thermo-mechanical transitions (i.e., T_onset_, T_peak_ and G’_max_), SV, and crumb texture were relevant factors contributing to the differentiation among samples. From a compositional standpoint, a determining role was observed for β-glucan contents in the dough on SV and bread crumb texture (Figure 7 and Figure 8). On the contrary, phenolics content (i.e., total phenolics and bound phenolics) only contributed to the differentiation of wheat bran (NWB and RWB)- and IWBF-enriched samples but did not show any direct contribution to bread quality, despite often being indicated as a main factor [11,57]. The negative correlation between SV and β-glucan content supported the results observed on the effect of oat bran and its fractions on gluten microstructure. Earlier, ref. [58] suggested that gluten aggregation during mixing depends on the viscosity of the water phase as affected by WEAX, rather than on interactions with ferulic acid. Gluten aggregation decreases with an increase in WEAX concentration and/or Mw [58,59]. Similarly, the behaviour of β-glucan in dough systems is governed by concentration and Mw, both affecting viscosity [60]. β-glucans are particularly effective in increasing viscosity due to their linear structure, high Mw, and their large number of hydroxyl groups [61], thus changing water distribution patterns in dough with less bulk water [37]. Overall, the viscosity of the water phase is a measure of solvent quality, as it is inversely related to its plasticizing ability with respect to biopolymers such as proteins and starches [13,16,62]. Consequently, an increase in β-glucans can negatively affect gluten development and aggregation [37,63] due to reduced hydrogen bonding interactions with the solvent. Additionally, direct hydrogen bond interactions between β-glucans and gluten can also contribute to changes in structural rearrangements [12]. As these mechanisms may be predominant for the soluble β-glucans, it can be suggested that the observed effects of total β-glucans on bread quality are controlled by the hydrogen bonding interaction of the soluble β-glucans and the water retention by the insoluble β-glucans, which hinder gluten hydration and structural rearrangements optimal for breadmaking [10,38].

The increase in cohesiveness and resilience with β-glucans content suggested that their effect may not be limited to gluten only. Crumb cohesiveness and resilience depend more on starch gelatinization and swelling than on gluten aggregation [56]. Analysis of DTMA and compositional data revealed that β-glucans content negatively correlated with the maximum gel strength G′_max_ at T_peak_ (Figure 7; R^2^ = 0.83, *p* < 0.05), which has been associated with the maximum in starch swelling [33,61]. In agreement with these observations, we have recently shown that the starch paste viscosity associated with gelatinization and swelling decreases with an increase in Mw and/or concentration of the fibres present [13]. However, cohesiveness and resilience should be negatively affected by inhibition of starch swelling and amylose leaching [32,64]. Most likely, these adverse effects were compensated for by the ability of the increasing β-glucans to form cross-links with the starch via hydrogen bonding, thus reinforcing the elastic properties of the starch gel network [65,66].

## 5. Conclusions

This study highlighted the effect of brans (wheat and oat) and bran fractions (soluble and insoluble) with distinct chemical and physical characteristics on dough and bread properties. By a systematic comparison of the properties of each fraction and the entire brans, the study indicated that the interplay between water binding, mainly provided by the insoluble fraction, and the plasticizing properties of the soluble bran fraction control the detrimental effects on bread volume and texture. On the contrary, steric hindrance and gluten dilution seemed to play a minor role, as indicated by the use of an inert filler (glass beads). The soluble oat bran fraction (i.e., SOBF) reduced the plasticizing ability of the water phase, as it was richer in β-glucans and in solutes of higher average Mw compared with the soluble wheat fraction (i.e., SWBF), as indicated by chemical analysis and sorption behaviour. Consequently, SOBF showed larger detrimental effects on SV than SWBF. The β-glucan content was a determining factor that discriminated the effects of wheat and oat brans and their fractions on dough and bread properties. Oat brans negatively impacted gluten microstructure compared with wheat brans, and SV was significantly affected by β-glucan content. The study also indicated that starch gelatinization and swelling is affected by the bran fractions and their interactions with water, as suggested by DMTA data on thermal transitions (T_onset_ and T_peak_) and dough elastic behaviour during setting (G’_max_). Interactions between starch and β-glucans via cross-linking during cooling may explain the observed increase in crumb cohesiveness and resilience with increasing β-glucan content.

## Figures and Tables

**Table 1:** Water absorption from farinograph tests with corresponding dough water content for the different doughs in the study.

	Water Absorption Properties	
Samples	Water Absorption ^a^ (Baker’s %)	Dough Water Content ^b^ (%wb)
Control (CB)	55.9	44.9
Glass beads (GB)	46.6	39.8
Native wheat bran (NWB)	63.0	45.7
Reconstituted wheat bran (RWB)	64.6	46.4
Soluble wheat bran fraction (SWBF)	46.3	39.7
Insoluble wheat bran fraction (IWBF)	65.9	46.7
Native oat bran (NOB)	75.3	49.6
Reconstituted oat bran (ROB)	75.9	49.8
Soluble oat bran fraction (SOBF)	53.4	42.5
Insoluble oat bran fraction (IOBF)	68.8	47.7

^**a**^ Water absorption from farinograph tests. ^**b**^ Dough water content calculated considering the initial moisture of the flour/mixtures and the water added based on farinograph analysis.

**Table 2:** Chemical and physical properties of bran and bran fractions from wheat and oat (samples abbreviations are reported in Table 1). Composition is expressed as grams of substance per grams of dry matter. Different letters in the same row indicate statistically significant differences (*p* < 0.05).

	NWB	RWB	SWBF	IWBF	NOB	ROB	SOBF	IOBF
Chemical compositions								
Starch	31.6 ± 0.6d	25.6 ± 0.5c	2.4 ± 0.0a	42.9 ± 1.6f	35.9 ± 0.8e	35.7 ± 0.5e	6.4 ± 0.1b	41.2 ± 0.6f
Protein	19.7 ± 0.3a	19.4 ± 0.4a	30.4 ± 2.0c	16.7 ± 0.7a	23.8 ± 0.4b	23.4 ± 0.1b	24.7 ± 0.2b	23.8 ± 0.6b
TDF	35.0 ± 0.4ef	32.4 ± 0.2de	20.6 ± 0.9a	38.4 ± 1.8g	28.2 ± 1.4bc	30.0 ± 1.2cd	36.9 ± 0.1fg	26.3 ± 0.3b
Total AX	14.7 ± 0.3c	15.0 ± 0.1c	3.25 ± 0.2ab	16.3 ± 0.0c	5.0 ± 0.9b	5.2 ± 0.9b	1.4 ± 0.0a	5.4 ± 1.2b
WEAX	0.65 ± 0.00c	0.89 ± 0.00d	3.25 ± 0.2e	0.12 ± 0.0a	<0.1	<0.1	0.4 ± 0.0b	<0.1
β-glucan	2.3 ± 0.1a	2.3 ± 0.1a	1.9 ± 0.2a	2.3 ± 0.2a	15.7 ± 0.2c	16.0 ± 0.3c	33.7 ± 0.2d	13.0 ± 0.6b
Total phenolics *	0.47 ± 0.12b	0.67 ± 0.02bc	0.83 ± 0.06d	0.53 ± 0.01bc	0.22 ± 0.04a	0.25 ± 0.03a	0.68 ± 0.02cd	0.18 ± 0.01a
Free phenolics	0.11 ± 0.01ab	0.26 ± 0.00bc	0.49 ± 0.03d	0.12 ± 0.00ab	0.12 ± 0.00ab	0.11 ± 0.00ab	0.41 ± 0.01cd	0.05 ± 0.00a
Bound phenolics	0.32 ±0.03c	0.34 ±0.03c	0.06 ±0.00ab	0.39 ±0.00c	0.06 ± 0.05a	0.09 ± 0.01ab	0.14 ± 0.01b	0.11 ± 0.00ab
Conjugated free phenolics	0.05 ± 0.01ab	0.08 ± 0.01bc	0.27 ± 0.03d	0.02 ± 0.00a	0.05 ± 0.00ab	0.05 ± 0.01ab	0.13 ± 0.00c	0.02 ± 0.01a
Physical properties								
D_[4,3]_ ** (μm)	288.5 ± 6.0d	243.0 ± 1.4c	-	254.5 ± 5.0c	134.0 ±1.4a	241.0 ±1.4b	-	217.0 ± 4.9c
WBC (g/g dm)	2.27 ± 0.16a	2.30 ± 0.01a	-	2.74 ± 0.01a	4.79 ± 0.54b	2.39±0.42a	4.72 ± 1.14b	2.69 ± 0.18a
g_water_/g_solids_ at a_w_ = 0.95	0.44 ± 0.00c	0.49 ± 0.00d	1.35 ± 0.00f	0.35 ± 0.00b	0.33 ± 0.00ab	0.34 ± 0.00ab	0.60 ± 0.00e	0.30 ± 0.00a

* Phenolics are expressed as ferulic acid equivalents. ** Volume weighted mean diameter from particle size analysis
